# Effect of aliovalent bismuth substitution on structure and optical properties of CsSnBr_3_

**DOI:** 10.1038/s42004-023-00874-w

**Published:** 2023-04-19

**Authors:** Madhusudan Chaudhary, Abhoy Karmakar, Vidyanshu Mishra, Amit Bhattacharya, Dundappa Mumbaraddi, Arthur Mar, Vladimir K. Michaelis

**Affiliations:** Department of Chemistry, University of Alberta, Edmonton, Alberta T6G 2G2 Canada

**Keywords:** Solid-state NMR, Characterization and analytical techniques, Solid-state chemistry, Energy

## Abstract

Aliovalent substitution of the *B* component in *ABX*_3_ metal halides has often been proposed to modify the band gap and thus the photovoltaic properties, but details about the resulting structure have remained largely unknown. Here, we examine these effects in Bi-substituted CsSnBr_3_. Powder X-ray diffraction (XRD) and solid-state ^119^Sn, ^133^Cs and ^209^Bi nuclear magnetic resonance (NMR) spectroscopy were carried out to infer how Bi substitution changes the structure of these compounds. The cubic perovskite structure is preserved upon Bi-substitution, but with disorder in the *B* site occurring at the atomic level. Bi atoms are randomly distributed as they substitute for Sn atoms with no evidence of Bi segregation. The absorption edge in the optical spectra shifts from 1.8 to 1.2 eV upon Bi-substitution, maintaining a direct band gap according to electronic structure calculations. It is shown that Bi-substitution improves resistance to degradation by inhibiting the oxidation of Sn.

## Abstract (web summary)

All-inorganic lead-free halide perovskites are highly promising photovoltaic materials, but higher-valent substitution of the divalent metal in such structures is poorly investigated. Here, the authors study the effects of substituting tin with bismuth in CsSnBr_3_ and find that the parent perovskite structure is preserved, while the absorption edge shifts, and degradation resistance is increased.

## Introduction

Hybrid organic-inorganic halide semiconductors have emerged as attractive photovoltaic materials because of their ease of fabrication, low cost, and high power conversion efficiencies, now exceeding 25%^1–5^. Unresolved problems arising from the poor thermal and moisture stability of these hybrid compounds have led many to reconsider the all-inorganic perovskites *ABX*_3_ (*A* = Cs; *B* = Sn, Pb, and other divalent metals; *X* = Cl, Br, I) as alternative candidates^6–10^. For example, the phase stability is improved by partially substituting organic cations by even low concentrations of Cs and introducing a combination of halogens in (CH(NH_2_)_2_)_0.83_Cs_0.17_Pb(I_0.6_Br_0.4_)_3_^11^. There remains a strong impetus to develop completely lead-free alternatives, by substituting Pb with other divalent metals such as Sn, Ge, Mn, and Sr^12–16^. The Sn-containing compounds tend to have smaller band gaps (e.g., ∼1.3 eV for orthorhombic CsSnI_3_), which are ideally suited for photoabsorption applications; they are also amenable to elemental substitution, enabling control and improved performance of light emitting diodes. Unfortunately, they suffer from the same recurring problem of instability in which the ease of oxidation (Sn^2+^ to Sn^4+^) necessitates strategies to avoid exposure to ambient conditions, or to fabricate the compounds under a reducing atmosphere^17–25^.

Recently, there have been many reports of aliovalent substitution of the divalent metal, whether Sn or Pb, in these halide semiconductors with a trivalent metal such as Sb or Bi^26–33^. This substitution (sometimes inappropriately termed as “alloying”) has been claimed to provide markedly improved stability upon exposure to air and moisture, and to enable band gaps to be adjusted to desired values. None of these studies offered detailed structural characterization, because of the difficulty of distinguishing between Sn from Sb, or Pb from Bi, through conventional X-ray diffraction (XRD) methods. It is not clear if this substitution occurs with completely random mixing, or whether the samples are actually more heterogeneous than assumed (e.g., phase segregation, secondary phases, or nanodomains). Moreover, substitution with a higher valent element such as Bi may entail n-doping, creation of vacancies in the metal sites, or incorporation of interstitial atoms. Some of these proposals have been investigated by theoretical calculations, but to date, experimental evidence has been scant^34,35^.

In this study, we choose CsSnBr_3_ as a model system to examine the effects of Bi substitution. By combining powder XRD with solid-state nuclear magnetic resonance (NMR) spectroscopy, which reveals information about the local environments around the Cs and Sn atoms, we endeavour to gain a fuller picture of the short- and long-range structure of these compounds^36–50^. Optical spectra were collected, and electronic structure calculations were performed to reveal further insight on the Bi substitution.

## Results and discussion

### Bi substitution in CsSnBr_3_

**Fig. 1:**
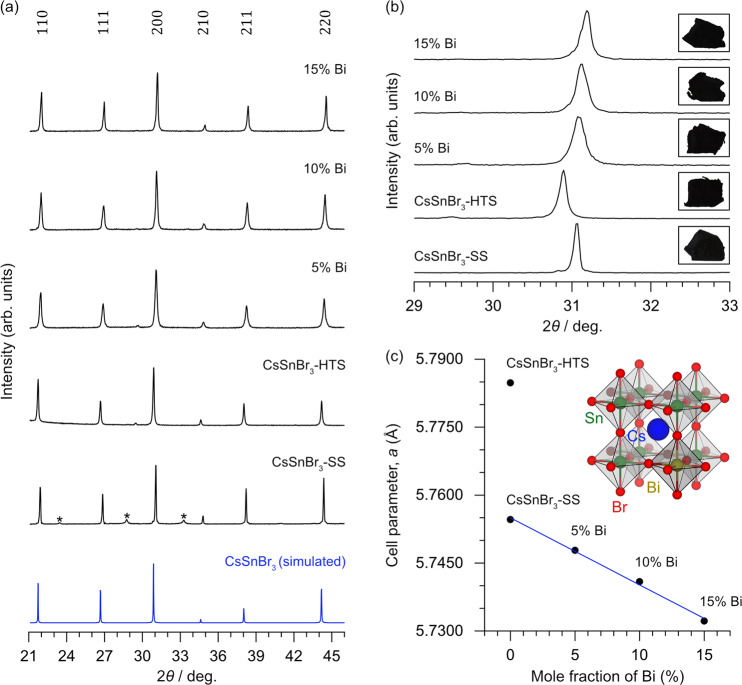
Long-range structure of CsSnBr_3_ and derivatives. **a** Powder XRD patterns for CsSnBr_3_ and Bi-substituted compounds. Trace secondary phases (*, Cs_2_SnBr_6_). **b** Comparison of 200 peaks showing shift to higher angles with greater Bi substitution (inset displays the colour of as synthesized compounds). **c** Evolution of cubic cell parameter *a* with Bi substitution.

Samples of CsSnBr_3_ were prepared by reaction of CsBr and SnBr_2_ in solution or directly at high temperature (600 °C for 5 h). Substitution of up to 15% Bi, through addition of BiBr_3_ using a high-temperature route (450 °C for 5 h), was successful. The powder XRD patterns confirmed the presence of the ideal cubic perovskite phase (space group ${Pm}\bar{3}m$) in all samples (Fig. 1a). All CsSnBr_3_ samples were slightly prone to oxidation, as evidenced by the formation of small amounts of Cs_2_SnBr_6_ if no special precautions were taken to avoid exposure to ambient conditions. In contrast, freshly prepared Bi-substituted samples were essentially single-phase and were more resistant to oxidation, as discussed later.

It is interesting that the XRD patterns for the CsSnBr_3_-HTS (high temperature synthesis) and CsSnBr_3_-SS (solvent synthesis) samples are slightly shifted relative to each other (Fig. 1b). As discussed later, we speculate that this difference is connected to the effect of a stereochemically active lone pair on Sn atoms^51^, which may be suppressed in the solution-prepared sample. The cubic cell parameter (5.7848(6) Å) for the CsSnBr_3_-HTS sample is consistent with literature values of similarly prepared samples (5.79–5.80 Å), whereas that for the CsSnBr_3_-SS sample is shorter (5.7546(2) Å)^51^. Starting from either case, the peaks in the XRD patterns shift to higher angles with increasing content of Bi (Fig. 1b). Although no reliable value of the ionic radius of Sn^2+^ has been reported, inspection of structurally characterized compounds containing formally trivalent Bi or divalent Sn surrounded octahedrally by Br atoms shows that average Bi–Br distances tend to be slightly shorter (2.82–2.88 Å in BiBr_3_, Rb_3_BiBr_6_, Cs_3_Bi_2_Br_9_, and Cs_2_AgBiBr_6_) than Sn–Br distances (2.90–2.92 Å in CsSnBr_3_)^52^. Accordingly, the cubic cell parameter refined from the XRD patterns gradually shortens with greater Bi substitution (Fig. 1c and Table 1). The samples consist of crystallites on the order of 10–100 μm dimensions (Fig. S1), and energy-dispersive X-ray spectroscopy (EDX) analyses showed the presence of the expected elements in the samples (Table 1 and Table S1, Figs. S2–S6). In particular, the relative amounts of Sn and Bi are close to the nominally loaded compositions.

**Table 1:** Nominal compositions, EDX analyses, and refined cubic cell parameters for all compounds.

Sample	Nominal compositions	Sn:Bi ratio (%) from EDX analysis	Cell parameter, *a* (Å)
CsSnBr_3_-SS	CsSnBr_3_	–	5.7546(2)
CsSnBr_3_-HTS	CsSnBr_3_	–	5.7848(6)
5% Bi	CsSn_0.95_Bi_0.05_Br_3.05_	94.9:5.1	5.7478(6)
10% Bi	CsSn_0.90_Bi_0.10_Br_3.10_	90.1:9.9	5.7409(5)
15% Bi	CsSn_0.85_Bi_0.15_Br_3.15_	85.7:14.3	5.7322(8)

### Influence of Bi substitution on local structure

Many competing hypotheses can be invoked for the structural effects of aliovalent substitution in halide semiconductors, *ABX*_3_. The most parsimonious model is that Bi atoms occupy the *B* site in a random fashion, but more exotic mechanisms such as placing Bi atoms within interstitial sites or forming Bi–Bi dimers have also been proposed^53–56^. When trivalent Bi replaces divalent Sn in CsSnBr_3_, a charge compensation mechanism could involve formation of cation vacancies (☐) in the *B* site, in a formulation such as ${{{{{\rm{Cs}}}}}}{{{{{{\rm{Sn}}}}}}}_{1-\tfrac{3}{2}x}{{{{{{\rm{Bi}}}}}}}_{x}{{{\square }}}_{x/2}{{{{{{\rm{Br}}}}}}}_{3}$, or perhaps in the *A* site. Alternatively, additional Br atoms in the form of anion interstitials could be proposed. NMR spectroscopy involving the accessible nuclei ^133^Cs and ^119^Sn may help shed light on which of these proposals is supported.

**Fig. 2:**
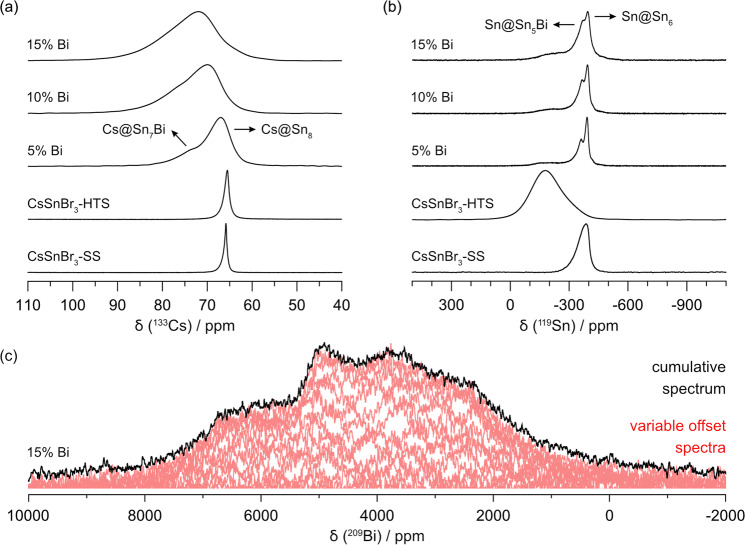
Multinuclear magnetic resonance of CsSnBr_3_ and Bi-substituted materials. **a** ^133^Cs and **b** ^119^Sn MAS NMR spectra (B_0_ = 11.75 T, ω_r_/2π = 14 kHz) for CsSnBr_3_ and Bi-substituted compounds. **c** Non-spinning ^209^Bi NMR spectrum for the 15% Bi-substituted compound (B_0_ = 11.75 T).

In CsSnBr_3_, the Cs atoms are surrounded in the first coordination sphere by twelve Br atoms in a cuboctahedral geometry and in the second coordination sphere by eight Sn atoms in cubic geometry. Substitution of Bi for Sn is expected to disrupt this highly symmetric environment, which can be probed by ^133^Cs NMR spectroscopy (Fig. 2a). CsSnBr_3_ shows a single isotropic resonance which is sharper for the sample prepared in solution (δ_iso_ = 65.8 ppm; full width at half-maximum, FWHM = 47 Hz) than the one prepared at high temperature (δ_iso_ = 65.6 ppm; FWHM = 82 Hz). This distinction has been noted previously^57^. It can be hypothesized that the sharper resonance in the solution-synthesized sample arises from fewer defects in the Br sites because of the Br-rich environment offered by the aqueous HBr medium.

At the levels of Bi substitution, up to 15%, two types of environments in the second coordination sphere around Cs may now be anticipated: Cs@Sn_8_ and Cs@Sn_7_Bi. Two peaks appear to be resolved in the ^133^Cs NMR spectra for the 5% Bi sample, but they merge as a broadened manifold for the 10% and 15% Bi samples. The lower frequency resonance can be assigned to the Cs@Sn_8_ environment; it shifts gradually to higher frequency (67 ppm for 5% Bi, 70 ppm for 10% Bi, and 72 ppm for 15% Bi), similar to observations made on other Cs-containing halides^13,58–60^. The higher frequency resonance can be assigned to the Cs@Sn_7_Bi environment, identified as a shoulder at 73 ppm for 5% Bi and at 76 ppm for 10% Bi samples, but not resolvable for the 15% Bi sample. The relative peak area of this resonance also becomes larger, consistent with greater Bi substitution. The occurrence of *B* site vacancies would also give rise to additional environments such as Cs@Sn_7_□, entailing local structural distortions that contribute to the line broadening. In any event, these resonances can be confidently attributed to the cubic perovskite phase, because the ^133^Cs peaks belonging to the potential secondary phases present occur at very different frequencies (261 ppm for CsBr, 227 ppm for CsSn_2_Br_5_, and 115 ppm for Cs_2_SnBr_6_) (Fig. S7)^13,37,57,61^.

The ^119^Sn NMR chemical shift range spans 1000’s of ppm (−5000 to 8000 ppm) when considering various oxidation states of tin compounds^44,62–65^. As such, ^119^Sn NMR lineshape, width and chemical shifts can be heavily influenced by local structural (dis)order and dynamics^13,36,44,61,62,66–68^. The Sn atoms are surrounded by six Br atoms in an ideal octahedral geometry in CsSnBr_3_. Upon Bi substitution, the environment around the Sn atoms changes in the third coordination sphere, consisting of six *B* sites also in an octahedral geometry at a distance equal to the unit cell length. These environments were probed by ^119^Sn NMR spectroscopy (Fig. 2b). For the parent CsSnBr_3_ compounds, a single asymmetric resonance is observed whose chemical shift and lineshape are highly sensitive to the synthetic method. This resonance broadens considerably on proceeding from the sample prepared in solution (δ_cgs_ = −386 ppm, FWHM = 12.4 kHz) to the one prepared at high temperature (δ_cgs_ = −179 ppm, FWHM = 34.7 kHz). For the solution-synthesized sample, a portion of the broadening is associated with ^119^Sn–^79/81^Br *J*-coupling, similar to related interactions in CsPbCl_3_ and MAPbCl_3_^41,45,69^. For the high-temperature-synthesized samples, the enhanced broadening could be due to formation of vacancies in the structure resulting from oxidation of Sn^2+^ to Sn^4+^. A more probable explanation is that this high-temperature synthetic route permits local distortions to develop around Sn atoms, which deviate from their ideal octahedral sites to energetically more favourable off-centre positions, as suggested recently by others in relation to the activity of the stereochemical lone pair, whereas the solution route does not lead to such distortions because it is performed at room temperature^51,70^. Resonances for potential Sn-containing impurity phases occur at much lower frequencies (−640 ppm for SnBr_2_, −659 ppm for SnBr_4_, and −1964 ppm for Cs_2_SnBr_6_)^37,44,61,62^.

When Bi is substituted within CsSnBr_3_, these Sn resonances become sharp again, perhaps by suppressing the Sn oxidation pathway or by favouring the centred octahedral positions (i.e., reducing lone-pair activity). Two peaks can be resolved, which can be assigned to the differing environments in the third coordination sphere: Sn@Sn_6_ at lower frequency and Sn@Sn_5_Bi at higher frequency. Both peaks shift slightly with greater Bi substitution, at δ_cgs_ of −391 and −360 ppm for 5% Bi, −393 and −366 ppm for 10% Bi, and −396 and −372 ppm for 15% Bi. This is consistent with increased covalent character in Sn–Br bonds as the unit cell decreases. The relative intensity of the Sn@Sn_5_Bi peak also increases as more Bi is introduced. A very broad ^119^Sn resonance with low intensity centred near −200 ppm appears reproducibly for all these Bi-substituted samples and cannot be assigned to any secondary phases^37,44,61,62^. They could be due to *B* site vacancies, as discussed above, resulting in additional environments Sn@Sn_5_□. Nevertheless, this feature is not prominent, and does not support more exotic structural models, such as Bi clustering, which would give rise to much more drastic changes in the local environment.

Although challenging, ^209^Bi NMR is gaining attention as a sensitive method to probe solids^71,72^. For example, in double perovskites, it has been used to examine ion substitution in Mn-Cs_2_NaBiCl_6_^73^, Cs_2_(Bi_1–*x*_In_*x*_)AgCl_6_^74^, and Cs_2_AgBiBr_6_^75^; in these cases, the [BiX_6_]^3−^ octahedra are rather symmetrical, which limits the broadening induced by quadrupolar coupling. The 15% Bi-substituted sample was chosen for study because it offers the highest sensitivity. Figure 2c displays a resonance that is clearly dominated by second-order quadrupolar broadening. The complex lineshape could not be simulated by a single environment around Bi atoms. Instead, the Bi atoms are likely experiencing multiple chemical environments, each distinct, involving different numbers of neighbouring Sn atoms or B-site vacancies. The electric field gradient about Bi is non-spherical, which is expected due to the potential occurrence of lone-pair effects, vacancies, and strain. Unfortunately, the long acquisition times (7 days) inhibit further exploration at multiple field strengths.

### Stability to moisture

**Fig. 3:**
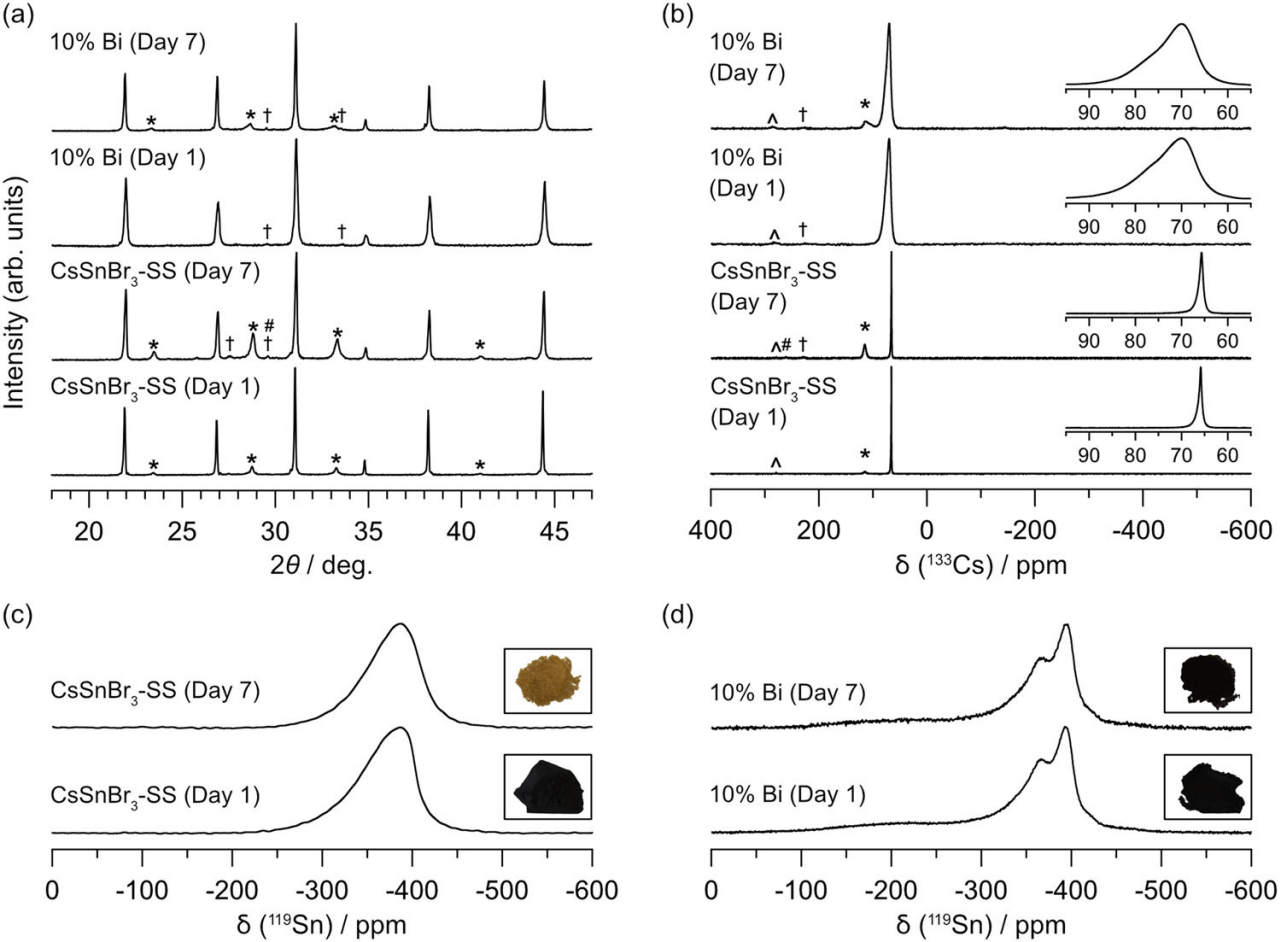
Exploration of degradation resistance via XRD and NMR. **a** Powder XRD patterns and **b** ^133^Cs MAS NMR spectra (B_0_ = 11.75 T, ω_r_/2π = 14 kHz) for solution-synthesized CsSnBr_3_ and high temperature synthesized 10% Bi-substituted samples, before and after exposure to 65% relative humidity. Secondary phases include Cs_2_SnBr_6_ (*), CsBr (#), and CsSn_2_Br_5_ (†). Spinning side bands are marked by carets (^). **c**, **d** ^119^Sn MAS NMR spectra (B_0_ = 11.75 T, ω_r_/2π = 14 kHz) for solution-synthesized CsSnBr_3_ and high temperature synthesized 10% Bi-substituted samples, respectively. Day 1 (before) and Day 7 (after) exposure to a 65% relative humidity. Photo insets are of powdered samples before and after exposure to humidity.

Powder XRD patterns and ^133^Cs/^119^Sn NMR spectra were collected for the solution-synthesized CsSnBr_3_ and 10% Bi-substituted samples after they were exposed to a relative humidity of 65% for 7 days (Fig. 3)^76^. The powder XRD patterns (Fig. 3a) show that the CsSnBr_3_ sample degraded to form considerable amounts of Cs_2_SnBr_6_, as well as small amounts of CsBr and CsSn_2_Br_5_, whereas the 10% Bi-substituted sample suffered less degradation. The ^133^Cs NMR spectra corroborate these results, showing increased peak intensities for these degradation by-products (115 ppm for Cs_2_SnBr_6_, 261 ppm for CsBr, 227 ppm for CsSn_2_Br_5_) and a broadened peak width for the main resonance at 65.8 ppm (FWHM of 66 Hz) for the CsSnBr_3_ sample (Fig. 3b). As estimated from relative peak areas (acquired using 10 min recycle delays), the amount of Cs_2_SnBr_6_ increased more than threefold, from 10% on day 1 to 36% on day 7, but the amounts of CsBr and CsSn_2_Br_5_ remain small (<1%). In contrast, for the 10% Bi-substituted sample, the amount of Cs_2_SnBr_6_ increased only to 8%, while the amount of CsSn_2_Br_5_ (<0.5%) present before exposure to moisture was unchanged. Finally, the ^133^Cs NMR resonances recorded are virtually identical before and after exposure to humidity for the 10% Bi-substituted compound. Figure 3c shows the ^119^Sn NMR for the CsSnBr_3_-SS sample before and after exposure to humidity. The ^119^Sn resonances have the same δ_cgs_ = −386 ppm, but some broadening is observed (12.4 to 12.6 kHz). The colour of the CsSnBr_3_-SS sample also changes from black to yellow after exposure. In contrast, the ^119^Sn resonances (Fig. 3d) and colour (inset) remains nearly identical for the 10% Bi-substituted sample after being exposed to a 65% relative humidity for 7 days. Scanning electron microscope (SEM) images and elemental maps are provided in Figs. S11–S13.

### Optical spectra and electronic structure

**Fig. 4:**
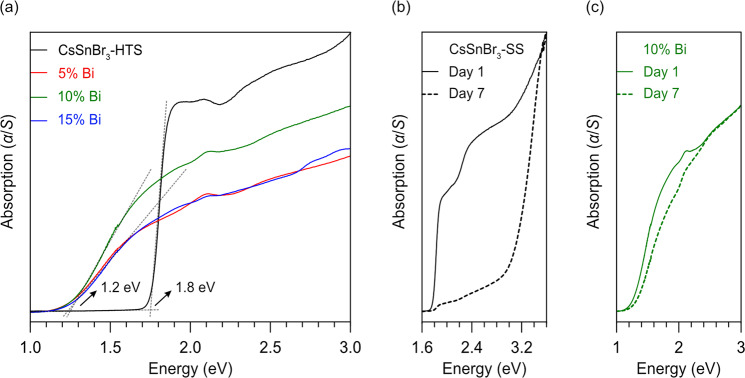
Optical properties for CsSnBr_3_ and Bi-substituted materials. Optical diffuse reflectance spectra for **a** CsSnBr_3_ (synthesized at high temperature) and Bi-substituted samples, **b** CsSnBr_3_ (synthesized in solvent), and **c** 10% Bi-substituted compound (synthesized at high temperature) before and after exposure to a 65% relative humidity.

CsSnBr_3_ exhibits a strong absorption edge in its optical spectrum (Fig. 4a), corresponding to a direct band gap of 1.8 eV, consistent with literature values (which range from 1.7 to 1.9 eV, depending on the form of the sample, although these are often reported with more precision than warranted)^77–79^. The spectrum shown is for the CsSnBr_3_-HTS sample, but the same result is obtained for the CsSnBr_3_-SS sample (Fig. 4b). The absorption edge shifts to 1.2 eV upon substitution with 5% Bi and does not change with higher amounts of Bi. This observation is similar to the cases of CsSnI_3_, CsPbBr_3_, and MAPbBr_3_, in which substitution of as little as 0.1% Bi abruptly shifts the absorption edge to lower energy by 0.2–0.4 eV^31,32,80–82^. The claims that this aliovalent substitution leads to a band gap narrowing have been criticized as a misinterpretation, and instead localized defect states appear in the midgap region below the conduction band, which leads to a broadening of the absorption edge in the optical spectra^83–87^. The absorption edge shifts to higher energy for the CsSnBr_3_-SS sample upon exposure to humidity, presumably because of degradation (Fig. 4b), whereas it is little affected for the 10% Bi-substituted sample (Fig. 4c).

**Fig. 5:**
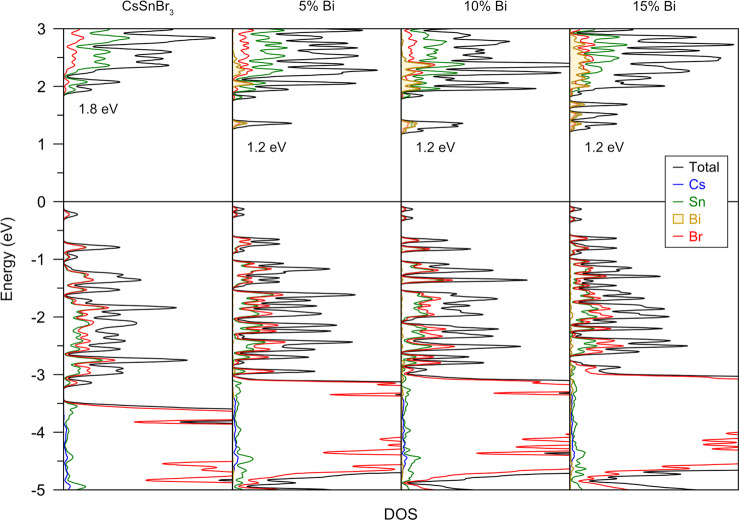
Electronic property calculations. DOS curves for CsSnBr_3_ and Bi-substituted models, from calculations using HSE06 functionals.

Various superstructure models of Bi-substituted CsSnBr_3_ were considered for electronic structure calculations. With the standard PBE functionals used in these calculations, the intrinsic band gap of 0.64 eV in CsSnBr_3_ is significantly underestimated compared to the experimentally observed value of 1.8 eV (Fig. S8). However, the main goal here was to examine the effect of Bi substitution. Accordingly, Bi-based impurity levels appear in the density of states (DOS), and the important observation is that they remain pinned below the conduction band with increasing Bi substitution, in agreement with the optical spectra. Although there are small changes in the cubic cell parameter, they are insufficient to cause any narrowing of the intrinsic gap between the valence band maximum and the conduction band minimum, both of which remain at the Brillouin zone centre (Fig. S9). The rest of the features are consistent with previous calculations on CsSnBr_3_, with the valence band dominated by anion states (Br), and the conduction band dominated by cation states (Sn, and at much higher energies, Cs). When the calculations are repeated using more accurate hybrid HSE06 functionals (Fig. 5), the calculated band gap of 1.8 eV in CsSnBr_3_ agrees well with experiment, and the Bi-based impurity levels are found at 0.6 eV below the conduction band, in agreement with the absorption edges of 1.2 eV in the optical spectra.

As depicted in ELF plots, strong covalent bonding interactions are evident between Sn and Br atoms in CsSnBr_3_ (Fig. S10), and the Bader charges are 0.9+ for Cs, 1.1+ for Sn, and 0.7− for Br. For the Bi-substituted models, there is a slight increase in electron density around the Bi atoms, but the Bader charges are not significantly changed, 0.9+ for Cs, 1.0+ to 1.1+ for Sn, 1.4 for Bi, and 0.6− to 0.7− for Br.

## Conclusions

The structure and optical properties of CsSnBr_3_ are influenced by synthetic approach and aliovalent Bi substitution, which can be achieved up to 15% with minimal side products. The long-range cubic perovskite structure is retained, but Bi substitution into the octahedral Sn site tends to suppress distortions arising from stereochemically active lone pairs in samples synthesized at high temperature. No evidence is seen to support proposals of Bi atoms entering interstitial sites or forming Bi–Bi dimers, suggesting that the perovskite structure can tolerate loadings up to 15%. The Bi-substituted compounds are significantly more resistant to oxidation of Sn^2+^ to Sn^4+^ when exposed to moisture. The absorption edge shifts from 1.8 eV in CsSnBr_3_ to 1.2 eV in the Bi-substituted compounds because defect states appear at 0.6 eV below the conduction band, as supported by quantum chemical calculations. These results suggest that aliovalent substitution is a promising approach to develop pathways for inexpensive and stable lead-free perovskites for photovoltaic applications.

## Methods

### Synthesis

The starting materials CsBr (Alfa Aesar, 99.9%), SnBr_2_ (Alfa Aesar, 99.2%), and BiBr_3_ (Alfa Aesar, 99%) were handled in an argon-filled glovebox with levels of <1 ppm H_2_O and O_2_ levels being maintained.

CsSnBr_3_ was prepared from 0.5 g equimolar mixtures of CsBr and SnBr_2_ which were ground using an agate mortar and pestle within the glovebox, and then reacted either in solution or directly at high temperature. In the first route, the mixture was dissolved in an aqueous solution consisting of 4.5 mL of HBr (Thermo Scientific, 48% wt/wt aq. soln.) and 0.5 mL H_3_PO_2_ (Sigma-Aldrich, 50% wt/wt aq. soln.), accompanied by a continuous flow of nitrogen gas. The solution was stirred for 20 min. The resulting precipitate was filtered, washed with isopropanol (Fisher, 99.5%), and dried under reduced pressure for 30 min. This solution-synthesized sample was labelled as “CsSnBr_3_-SS.” In the second route, the mixture was cold-pressed into a pellet and placed within a fused-silica tube, which was evacuated (<10^−3^ Torr) and sealed. The tube was placed in a furnace where it was heated to 600 °C in 12 h, held at this temperature for 5 h, and then cooled to room temperature over 12 h. This high-temperature-synthesized sample was labelled as “CsSnBr_3_-HTS.”

Bi substitution in CsSnBr_3_ was attempted by combining CsBr, SnBr_2_, and BiBr_3_ in molar ratios of 1:(1 − *x*):*x*, where *x* = 0.05, 0.10, and 0.15. The mixtures were ground within the glovebox, sealed within evacuated fused-silica tubes, and heated to 450 °C in 9 h, held at this temperature for 5 h, and then cooled to room temperature over 9 h. They are labelled as 5%, 10%, and 15% Bi-substituted samples, with the understanding that these refer to the nominally loaded compositions. Higher level of Bi substitutions, above 15%, led to multiphase products.

### Powder XRD

Powder XRD patterns of ground samples were collected on a Bruker D8 Advance diffractometer equipped with a Cu radiation source (*K*α_1_ wavelength of 1.54056 Å) operated at 40 kV and 40 mA. Pawley refinements were performed, with the background modelled by a twelve-term polynomial function, using the TOPAS Academic software package^88^.

### FESEM and EDX analysis

Powdered samples were examined on a Zeiss Sigma 300 VP field-emission scanning electron microscope (FESEM) equipped with dual silicon drift detectors operated with an accelerating voltage of 15 kV. Elemental compositions were determined by EDX area analyses with acquisition times of 120 s.

### Solid-state NMR spectroscopy

The ^133^Cs, ^119^Sn, and ^209^Bi NMR experiments were performed on a Bruker Avance NEO 500 (B_0_ = 11.75 T) NMR spectrometer, equipped with a 4 mm double resonance H-X magic-angle spinning (MAS) Bruker NMR probe. Powdered samples were packed into 4 mm o.d. ZrO_2_ ceramic rotors which were sealed with Kel-F drive caps. The ^133^Cs MAS NMR spectra for all samples were acquired using a 1.5 µs (liquid, γB_1_/2π = 41.6 kHz) Bloch pulse, 16 co-added transients, ω_r_/2π = 14 kHz, and a recycle delay of 500 s. These spectra were referenced to 0 ppm using a CsNO_3_ (0.1 M) aqueous solution. For the CsSnBr_3_-SS sample, ^119^Sn MAS NMR spectra were acquired using a π/4 Bloch decay pulse (2 μs) with a recycle delay of 0.1 s for 10,240 co-added transients. For the CsSnBr_3_-HTS sample, ^119^Sn MAS NMR spectra were acquired using a rotor-synchronized Hahn echo pulse (π/2-τ-π-acq.) with 4 and 8 μs pulse widths (γB_1_/2π = 62.5 kHz), 0.1 s recycle delay, and 51,200 co-added transients. For the Bi-substituted samples, ^119^Sn MAS NMR spectra were acquired using a rotor-synchronized Hahn echo pulse (π/2-τ-π-acq.) with 4 and 8 μs pulse widths (γB_1_/2π = 62.5 kHz), 1.5 s recycle delay, and 20,480 co-added transients. All spectra employing a Hahn echo were acquired with the variable offset cumulative spectrum (VOCS) approach using 30–50 kHz offsets in 2–3 steps. These spectra were referenced to Me_4_Si (δ_iso_ = 0 ppm) by setting the isotropic peak of tetracyclohexyltin to −97.35 ppm. The ^209^Bi NMR experiments were acquired using a Hahn echo pulse sequence with 1.2 and 2.4 μs pulse widths employing the VOCS method. Each offset step was set to 50 kHz and employing 0.01 s recycle delay with 300,000 co-added transients. The ^209^Bi NMR data were referenced to 0 ppm using a saturated solution of Bi(NO_3_)_3_ in HNO_3_ and D_2_O.

### Phase stability tests

The solution-synthesized CsSnBr_3_ and 10% Bi-substituted samples were exposed to a relative humidity of 65 ± 5% within a home-built humidity chamber for 7 days^76^. Powder XRD patterns, ^133^Cs/^119^Sn NMR spectra and optical measurements were recollected after this treatment.

### Optical spectroscopy

Optical diffuse reflectance spectra of ground samples were collected on a Cary 5000 UV-vis-NIR spectrophotometer. An optical polytetrafluoroethylene disc (>98% reflectivity between 250 and 2200 nm) was used as a reflectance standard. The reflectance spectra were converted to optical absorption spectra using the Kubelka–Munk function, *F*(*R*) = α/*S* = (1 − *R*)^2^/2*R*, where α is the Kubelka–Munk absorption coefficient, *S* is the scattering coefficient, and *R* is the reflectance^89^.

### Electronic structure calculations

Various superstructure models were generated using the program Supercell (version 2.0) to accommodate Bi substitution within CsSnBr_3_, involving different distributions of Sn and Bi atoms, creation of vacancies at Cs or Sn sites, and permutations of atoms and vacancies^90^. The models with the lowest Coulomb energies were then chosen to calculate the electronic structure, using the projected augmented wave (PAW) method as implemented in the Vienna ab initio simulation package (VASP version 5.4.4)^91–93^. The generalized gradient approximation, as parameterized by Perdew, Burke, and Ernzerhof (PBE), was used to treat exchange and correlation^94–96^. The recommended standard PAW potentials Cs_sv, Sn_d, Br, and Bi_d were used, with the plane-wave basis cutoff energy set to 500 eV. An 8 × 8 × 3 gamma-centred *k*-point grid was used to sample the first Brillouin zone. The convergence criteria were set to |−2 × 10^−2^ | eV for ionic relaxation and 10^−8^ for electronic optimization. To correct the significant underestimation of the PBE band gap, additional calculations were performed in which an HSE06 functional (with a PBE:Hartree–Fock ratio of 42:58 for CsSnBr_3_ and 45:55 for Bi-substituted CsSnBr_3_) was applied. The first Brillouin zone was sampled using a 4 × 4 × 2 gamma-centred k-point grid, with the plane wave basis set cut-off energy set to 350 eV, and convergence criteria for electronic optimization set to 10^−6^ eV and |−2 × 10^−2^| eV for ionic relaxation. Spin-orbit interactions were not considered to reduce computational cost. Electron localization functions (ELF) and Bader charges were evaluated using the program LOBSTER (version 3.2.0)^97^.

## Supplementary information

Supplementary Information

## Data Availability

Additional data (SEM, Elemental mapping, NMR, ELF and PBE DOS plots) supporting the findings of this study are available within the Supplementary Information. Other data are available from the corresponding authors upon reasonable request.

## References

[1] NREL. *Interactive Best Research-Cell Efficiency Chart.* https://www.nrel.gov/pv/interactive-cell-efficiency.html (2022).

[2] Lin R (2022). All-perovskite tandem solar cells with improved grain surface passivation. Nature.

[3] Stoumpos CC, Kanatzidis MG (2016). Halide perovskites: poor man’s high-performance semiconductors. Adv. Mater..

[4] Manser JS, Christians JA, Kamat PV (2016). Intriguing optoelectronic properties of metal halide perovskites. Chem. Rev..

[5] Jeong J (2021). Pseudo-halide anion engineering for α-FAPbI_3_ perovskite solar cells. Nature.

[6] Wang D, Wright M, Elumalai NK, Uddin A (2016). Stability of perovskite solar cells. Sol. Energy Mater. Sol. Cells.

[7] Li M, Li H, Fu J, Liang T, Ma W (2020). Recent progress on the stability of perovskite solar cells in a humid environment. J. Phys. Chem. C.

[8] Liang J (2019). Defect-engineering-enabled high-efficiency all-inorganic perovskite solar cells. Adv. Mater..

[9] Li B, Long R, Xia Y, Mi Q (2018). All-inorganic perovskite CsSnBr_3_ as a thermally stable, free-carrier semiconductor. Angew. Chem. Int. Ed..

[10] Liang J (2016). All-inorganic perovskite solar cells. J. Am. Chem. Soc..

[11] McMeekin DP (2016). A mixed-cation lead mixed-halide perovskite absorber for tandem solar cells. Science.

[12] Kour R (2019). Potential substitutes for replacement of lead in perovskite solar cells: a review. Glob. Chall..

[13] Karmakar A, Bhattacharya A, Bernard GM, Mar A, Michaelis VK (2021). Revealing the local Sn and Pb arrangements in CsSn_x_Pb_1–x_Br_3_ perovskites with solid-state NMR spectroscopy. ACS Mater. Lett..

[14] Hooper RW (2022). Exploring structural nuances in germanium halide perovskites using solid-state ^73^Ge and ^133^Cs NMR spectroscopy. J. Phys. Chem. Lett..

[15] Zhang Q (2018). Perovskite solar cells: must lead be replaced—and can it be done?. Sci. Technol. Adv. Mater..

[16] Abate A (2017). Perovskite solar cells go lead free. Joule.

[17] Shum K, Tsatskina A (2016). Solar cells: stabilizing tin-based perovskites. Nat. Energy.

[18] Kumar MH (2014). Lead-free halide perovskite solar cells with high photocurrents realized through vacancy modulation. Adv. Mater..

[19] Chung I, Lee B, He J, Chang RPH, Kanatzidis MG (2012). All-solid-state dye-sensitized solar cells with high efficiency. Nature.

[20] Ye T (2021). Ambient-air-stable lead-free CsSnI_3_ solar cells with greater than 7.5% efficiency. J. Am. Chem. Soc..

[21] Lanzetta L (2021). Degradation mechanism of hybrid tin-based perovskite solar cells and the critical role of tin (IV) iodide. Nat. Commun..

[22] Lu J (2021). Dendritic CsSnI_3_ for efficient and flexible near-infrared perovskite light-emitting diodes. Adv. Mater..

[23] Touré A, Bouich A, Soucasse BM, Soro D (2023). Investigation of the optoelectronics properties and stability of formamidinium lead mixed halides perovskite. Opt. Mater..

[24] Doumbia Y, Bouich A, Soro D, Soucase BM (2022). Mixed halide head perovskites thin films: stability and growth investigation. Optik.

[25] Bouich, A. *Study and Characterization of Hybrid Perovskites and Copper-indium-gallium Selenide Thin Films for Tandem Solar Cells*. Universitat Politècnica de València (2021).

[26] Yin J, Ahmed GH, Bakr OM, Brédas J-L, Mohammed OF (2019). Unlocking the effect of trivalent metal doping in all-inorganic CsPbBr_3_ perovskite. ACS Energy Lett..

[27] Bera S (2019). Limiting heterovalent B-site doping in CsPbI_3_ nanocrystals: phase and optical stability. ACS Energy Lett..

[28] Zhang J, Yang L, Liu R, Chen L (2019). Stabilization of all-inorganic α-CsPbI_3_ perovskite by Bi or Sb doping. Mater. Res. Express.

[29] Khan MI (2022). Improving the structural, optical and photovoltaic properties of Sb- and Bi- Co-doped MAPbBr_3_ perovskite solar cell. Coatings.

[30] Marí-Guaita J, Bouich A, Marí B (2022). Stability improvement of methylammonium lead iodide perovskite thin films by bismuth doping. JOM.

[31] Lee M, Yoo B, Im J, Hyeon T, Chung I (2020). Electronic band engineering via MI_3_ (M = Sb, Bi) doping remarkably enhances the air stability of perovskite CsSnI_3_. ACS Appl. Energy Mater..

[32] Abdelhady AL (2016). Heterovalent dopant incorporation for bandgap and type engineering of perovskite crystals. J. Phys. Chem. Lett..

[33] Hu Y (2017). Bismuth incorporation stabilized α-CsPbI_3_ for fully inorganic perovskite solar cells. ACS Energy Lett..

[34] Mosconi E, Merabet B, Meggiolaro D, Zaoui A, De Angelis F (2018). First-principles modeling of bismuth doping in the MAPbI_3_ perovskite. J. Phys. Chem. C..

[35] Kang Y, Kang S, Han S (2019). Influence of Bi doping on physical properties of lead halide perovskites: a comparative first-principles study between CsPbI_3_ and CsPbBr_3_. Mater. Today Adv..

[36] Kubicki DJ, Stranks SD, Grey CP, Emsley L (2021). NMR spectroscopy probes microstructure, dynamics and doping of metal halide perovskites. Nat. Rev. Chem..

[37] Krajewska CJ (2021). Enhanced visible light absorption in layered Cs_3_Bi_2_Br_9_ through mixed-valence Sn(II)/Sn(IV) doping. Chem. Sci..

[38] Adeyemi AN (2022). Synthesis of SrTiO_3_ and Al-doped SrTiO_3_ via the deep eutectic solvent route. Mater. Adv..

[39] Raval P (2022). Understanding instability in formamidinium lead halide perovskites: kinetics of transformative reactions at grain and subgrain boundaries. ACS Energy Lett..

[40] Piveteau L, Morad V, Kovalenko MV (2020). Solid-state NMR and NQR spectroscopy of lead-halide perovskite materials. J. Am. Chem. Soc..

[41] Bernard GM (2018). Methylammonium cation dynamics in methylammonium lead halide perovskites: a solid-state NMR perspective. J. Phys. Chem. A.

[42] Grüninger H (2021). Microscopic (dis)order and dynamics of cations in mixed FA/MA lead halide perovskites. J. Phys. Chem. C.

[43] Moudrakovski IL (2021). Local dynamics in hybrid perovskites by solid-state NMR. Annu. Rep. NMR Spectrosc..

[44] Ha M (2020). Phase evolution in methylammonium tin halide perovskites with variable temperature solid-state ^119^Sn NMR spectroscopy. J. Phys. Chem. C..

[45] Bernard, G. M., Karmakar, A. & Michaelis, V. K. Solid-state NMR studies of halide perovskite materials with photoconversional potential, *Reference Module in Chemistry, Molecular Sciences and Chemical Engineering* (2021).

[46] Dahlman CJ, Kubicki DJ, Reddy GNM (2021). Interfaces in metal halide perovskites probed by solid-state NMR spectroscopy. J. Mater. Chem. A.

[47] Hooper RW, Sarkar D, Michaelis VK (2022). Bulk and nanoscale semiconducting materials: structural advances using solid-state NMR spectroscopy. Curr. Opin. Colloid Interface Sci..

[48] Fu P (2022). Short aromatic diammonium ions modulate distortions in 2D lead bromide perovskites for tunable white-light emission. Chem. Mater..

[49] Mishra A (2022). Interplay of kinetic and thermodynamic reaction control explains incorporation of dimethylammonium iodide into CsPbI_3_. ACS Energy Lett..

[50] Sarkar D (2022). Metal halide perovskite and perovskite-like materials through the lens of ultra-wideline ^35/37^Cl NMR spectroscopy. ACS Mater. Lett..

[51] Fabini DH (2016). Dynamic stereochemical activity of the Sn^2+^ lone pair in perovskite CsSnBr_3_. J. Am. Chem. Soc..

[52] Villars, P. & Cenzual, K. *Pearson’s Crystal Data: Crystal Structure Database for Inorganic Compounds (on DVD)* (ASM International, 2021).

[53] Mannodi-Kanakkithodi A (2019). Comprehensive computational study of partial lead substitution in methylammonium lead bromide. Chem. Mater..

[54] Lyons JL (2021). Effective donor dopants for lead halide perovskites. Chem. Mater..

[55] Pipitone C (2022). Bi^3+^ doping in 1D ((CH_3_)_3_SO)PbI_3_: a model for defect interactions in halide perovskites. J. Mater. Chem. C..

[56] Wang S, Huang M, Wu Y-N, Chen S (2022). Formation of Bi−Bi dimers in heavily Bi-doped lead halide perovskites: origin of carrier density saturation. Phys. Rev. Appl..

[57] Karmakar A (2021). Influence of hidden halogen mobility on local structure of CsSn(Cl_1−*x*_ Br_*x*_)_3_ mixed-halide perovskites by solid-state NMR. Chem. Sci..

[58] Karmakar A (2018). Mechanochemical synthesis of methylammonium lead mixed–halide perovskites: unraveling the solid-solution behavior using solid-state NMR. Chem. Mater..

[59] Kubicki DJ (2020). Halide mixing and phase segregation in Cs_2_AgBiX_6_ (X = Cl, Br, and I) double perovskites from cesium-133 solid-state NMR and optical spectroscopy. Chem. Mater..

[60] Xu T (2022). Simultaneous lattice engineering and defect control via cadmium incorporation for high-performance inorganic perovskite solar cells. Adv. Sci..

[61] Karmakar A (2021). Uncovering halogen mixing and octahedral dynamics in Cs_2_SnX_6_ by multinuclear magnetic resonance spectroscopy. Chem. Mater..

[62] Kubicki DJ (2020). Local structure and dynamics in methylammonium, formamidinium, and cesium tin(II) mixed-halide perovskites from ^119^Sn solid-state NMR. J. Am. Chem. Soc..

[63] Amornsakchai P, Apperley DC, Harris RK, Hodgkinson P, Waterfield PC (2004). Solid-state NMR studies of some tin(II) compounds. Solid State Nucl. Magn. Reson..

[64] Yeh H-MM, Geanangel RA (1981). ^119^Sn NMR spectra of tin(II) halides. Inorg. Chim. Acta.

[65] Sharp RR (1974). Field dependence of nuclear magnetic relaxation of ^119^Sn in SnCl_4_, SnBr_4_, and SnI_4_. J. Chem. Phys..

[66] Wang L (2015). Structure vs ^119^Sn NMR chemical shift in three-coordinated tin(II) complexes: experimental data and predictive DFT computations. Organometallics.

[67] Mitchell MR (2010). ^119^Sn MAS NMR and first-principles calculations for the investigation of disorder in stannate pyrochlores. Phys. Chem. Chem. Phys..

[68] Eichler BE, Phillips BL, Power PP, Augustine MP (2000). Solid-state and high-resolution liquid ^119^Sn NMR spectroscopy of some monomeric, two-coordinate low-valent tin compounds: very large chemical shift anisotropies. Inorg. Chem..

[69] Aebli M (2020). Lead-halide scalar couplings in ^207^Pb NMR of APbX_3_ perovskites (A = Cs, methylammonium, formamidinium; X = Cl, Br, I). Sci. Rep..

[70] Xie H (2020). All-inorganic halide perovskites as potential thermoelectric materials: dynamic cation off-centering induces ultralow thermal conductivity. J. Am. Chem. Soc..

[71] Hamaed H, Laschuk MW, Terskikh VV, Schurko RW (2009). Application of solid-state ^209^Bi NMR to the structural characterization of bismuth-containing materials. J. Am. Chem. Soc..

[72] Griffith KJ, Ding F, Flynn S (2021). Solid-state nuclear magnetic resonance of spin-9/2 nuclei ^115^In and ^209^Bi in functional inorganic complex oxides. Magn. Reson. Chem..

[73] Karmakar A (2023). Triangulating dopant-level Mn(II) insertion in a Cs_2_NaBiCl_6_ double perovskite using magnetic resonance spectroscopy. J. Am. Chem. Soc..

[74] Karmakar A, Bernard GM, Meldrum A, Oliynyk AO, Michaelis VK (2020). Tailorable indirect to direct band-gap double perovskites with bright white-light emission: decoding chemical structure using solid-state NMR. J. Am. Chem. Soc..

[75] Ji F (2020). Near-infrared light-responsive Cu-doped Cs_2_AgBiBr_6_. Adv. Funct. Mater..

[76] Askar AM, Bernard GM, Wiltshire B, Shankar K, Michaelis VK (2017). Multinuclear magnetic resonance tracking of hydro, thermal, and hydrothermal decomposition of CH_3_NH_3_PbI_3_. J. Phys. Chem. C..

[77] Gupta S, Bendikov T, Hodes G, Cahen D (2016). CsSnBr_3_, a lead-free halide perovskite for long-term solar cell application: insights on SnF_2_ addition. ACS Energy Lett..

[78] Díaz-Acosta CM (2021). ABX_3_ inorganic halide perovskites for solar cells: chemical and crystal structure stability. Matéria (Rio J.).

[79] Coduri M (2019). Band gap engineering in MASnBr_3_ and CsSnBr_3_ perovskites: mechanistic insights through the application of pressure. J. Phys. Chem. Lett..

[80] Amerling E (2021). A multi-dimensional perspective on electronic doping in metal halide perovskites. ACS Energy Lett..

[81] Begum R (2017). Engineering interfacial charge transfer in CsPbBr_3_ perovskite nanocrystals by heterovalent doping. J. Am. Chem. Soc..

[82] Miao X (2017). Air-stable CsPb_1−x_Bi_x_Br_3_ (0 ≤ x ≪ 1) perovskite crystals: optoelectronic and photostriction properties. J. Mater. Chem. C..

[83] Lozhkina OA (2018). Invalidity of band-gap engineering concept for Bi^3+^ heterovalent doping in CsPbBr_3_ halide perovskite. J. Phys. Chem. Lett..

[84] Nayak PK (2018). Impact of Bi^3+^ heterovalent doping in organic–inorganic metal halide perovskite crystals. J. Am. Chem. Soc..

[85] Meng R (2019). Understanding the impact of bismuth heterovalent doping on the structural and photophysical properties of CH_3_NH_3_PbBr_3_ halide perovskite crystals with near-IR photoluminescence. Chem. Eur. J..

[86] Yamada Y, Hoyano M, Akashi R, Oto K, Kanemitsu Y (2017). Impact of chemical doping on optical responses in bismuth-doped CH_3_NH_3_PbBr_3_ single crystals: carrier lifetime and photon recycling. J. Phys. Chem. Lett..

[87] Tang Z-K (2017). Enhanced optical absorption via cation doping hybrid lead iodine perovskites. Sci. Rep..

[88] Coelho, A. *TOPAS-Academic* (2007).

[89] Makuła P, Pacia M, Macyk W (2018). How to correctly determine the band gap energy of modified semiconductor photocatalysts based on UV–Vis spectra. J. Phys. Chem. Lett..

[90] Okhotnikov K, Charpentier T, Cadars S (2016). Supercell program: a combinatorial structure-generation approach for the local-level modeling of atomic substitutions and partial occupancies in crystals. J. Cheminform..

[91] Kresse G, Furthmüller J (1996). Efficient iterative schemes for ab initio total-energy calculations using a plane-wave basis set. Phys. Rev. B.

[92] Kresse G, Joubert D (1999). From ultrasoft pseudopotentials to the projector augmented-wave method. Phys. Rev. B.

[93] Blöchl PE (1994). Projector augmented-wave method. Phys. Rev. B.

[94] Perdew JP, Burke K, Ernzerhof M (1996). Generalized gradient approximation made simple. Phys. Rev. Lett..

[95] Hohenberg P, Kohn W (1964). Inhomogeneous electron gas. Phys. Rev..

[96] Kohn W, Sham LJ (1965). Self-consistent equations including exchange and correlation effects. Phys. Rev..

[97] Maintz S, Deringer VL, Tchougréeff AL, Dronskowski R (2016). LOBSTER: a tool to extract chemical bonding from plane-wave based DFT. J. Comput. Chem..

