# Supplementary material for: Effect of aliovalent bismuth substitution on structure and optical properties of CsSnBr3
Source: Commun Chem. 2023 Apr 19;6:75. doi: 10.1038/s42004-023-00874-w (PMC10115781; doi:10.1038/s42004-023-00874-w)
Supplement: Supplementary file 1 — Supplementary Information [file 42004_2023_874_MOESM1_ESM.pdf]

Supplemental Information

Effect of aliovalent bismuth substitution on structure and optical properties of $CsSnBr_3$

Madhusudan Chaudhary, Abhoy Karmakar, Vidyanshu Mishra, Amit Bhattacharya, Dundappa Mumbaraddi, Arthur Mar, and Vladimir K. Michaelis*

Department of Chemistry, University of Alberta, Edmonton, Alberta T6G 2G2, Canada

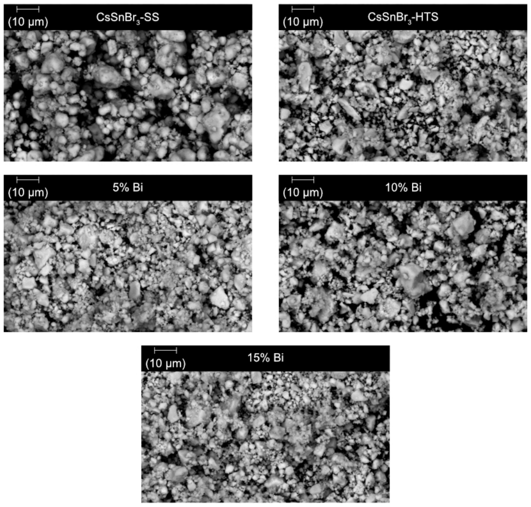

Figure S1. SEM images of $CsSnBr_3$ and Bi-substituted compounds.

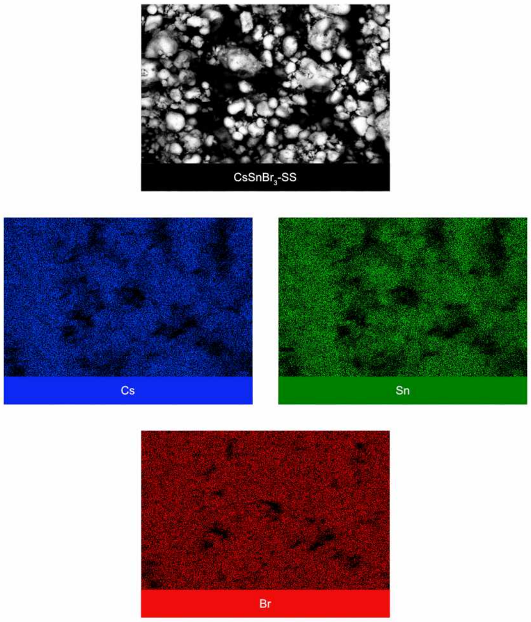

Figure S2. Elemental mapping of $CsSnBr_3$-SS.

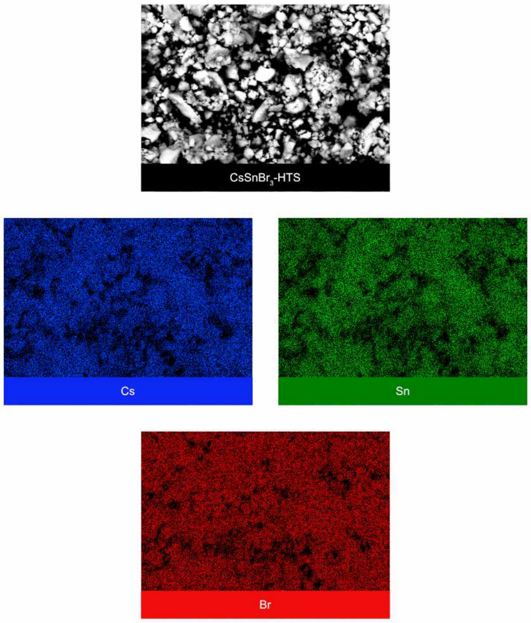

Figure S3. Elemental mapping of $CsSnBr_3$-HTS.

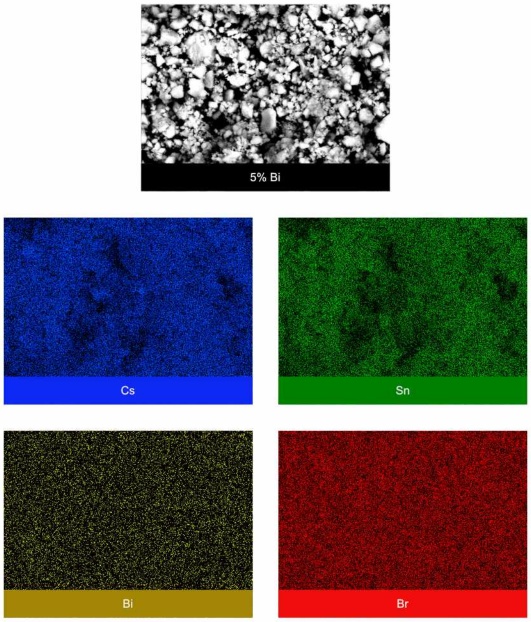

Figure S4. Elemental mapping of 5% Bi-substituted compound.

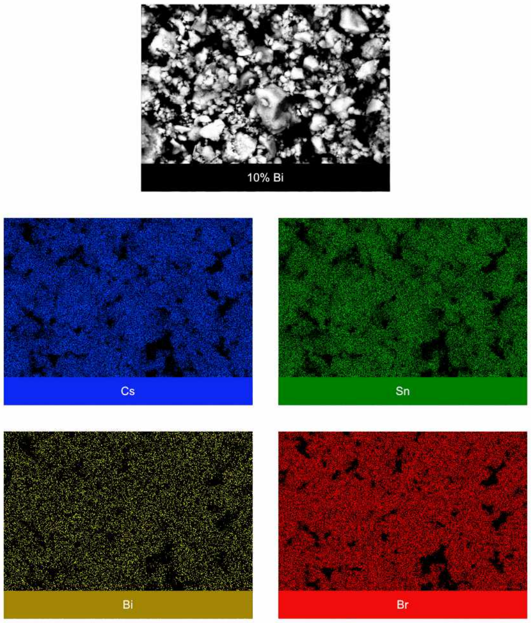

Figure S5. Elemental mapping of 10% Bi-substituted compound.

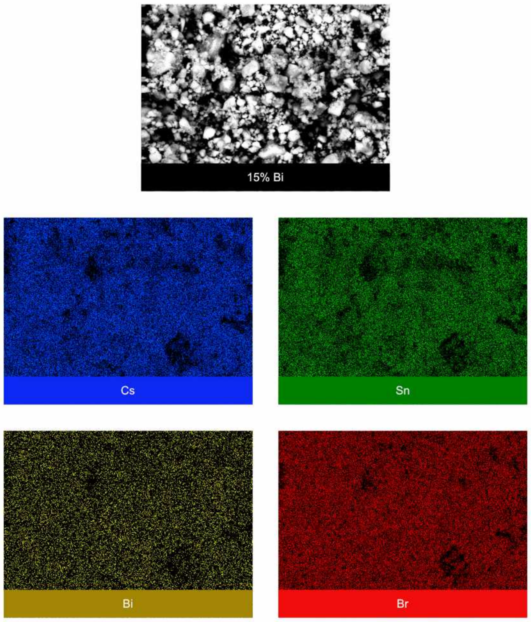

Figure S6. Elemental mapping of 15% Bi-substituted compound.

Table S1. Nominal compositions and EDX analyses of $CsSnBr_3$ and Bi-substituted compounds.

Sample	Cs atom (%)		Sn atom (%)		Bi atom (%)		Br atom (%)	
	Nominal	EDX analysis	Nominal	EDX analysis	Nominal	EDX analysis	Nominal	EDX analysis
$CsSnBr_3$-SS	20	21	20	20	0	0	60	59
$CsSnBr_3$-HTS	20	21	20	21	0	0	60	58
5%Bi	19.8	20	18.81	17	0.99	1	60.4	62
10%Bi	19.61	20	17.65	16	1.96	2	60.78	62
15%Bi	19.42	20	16.5	15	2.91	3	61.17	62

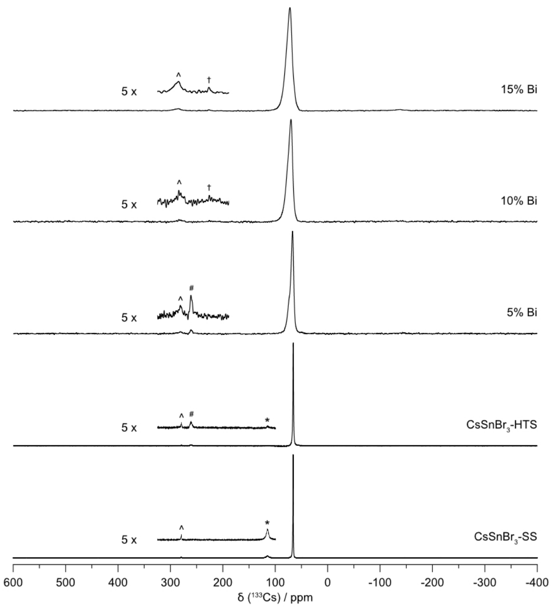

Figure S7. ^{133}Cs MAS NMR spectra (B_0 = 11.75 T, $\omega_r/2\pi$ = 14 kHz) for $CsSnBr_3$ and Bi-substituted samples. Trace secondary phases include Cs_2SnBr_6 (asterisks, *), CsBr (hash marks, #), and $CsSn_2Br_5$ (daggers, †). Spinning side bands are marked by carets, ^.

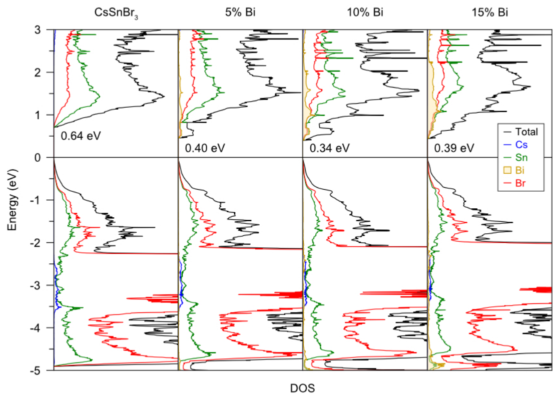

Figure S8. PBE DOS curves for $CsSnBr_3$ and Bi-substituted models.

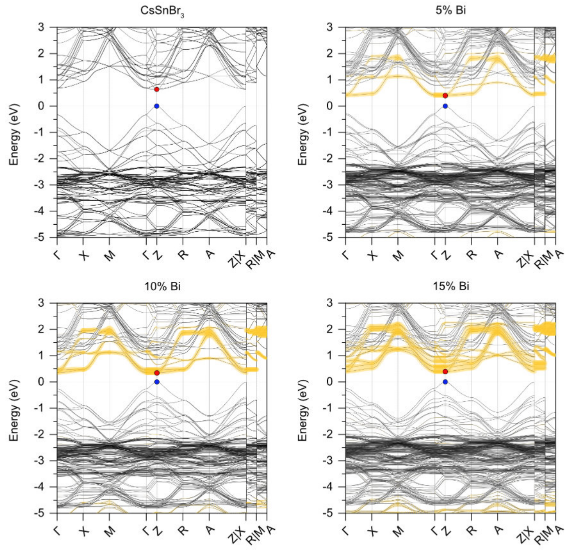

Figure S9. PBE band dispersion plots for $CsSnBr_3$ and Bi-substituted models. The contributions of Bi-based states are highlighted in yellow. The valence band maxima are marked by blue circles and the conduction band minima by red circles.

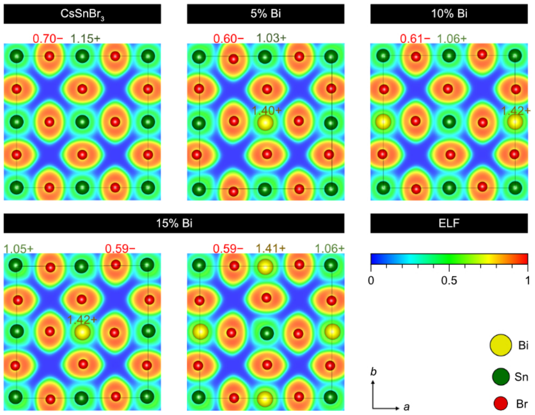

Figure S10. ELF plots and Bader charges for $CsSnBr_3$ and Bi-substituted models, from calculations using HSE06 functionals.

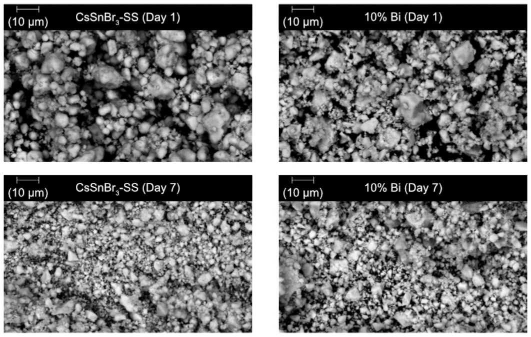

Figure S11. SEM images of $CsSnBr_3$ and 10% Bi-substituted compound, before (Day 1) and after (Day 7) exposure to 65% relative humidity.

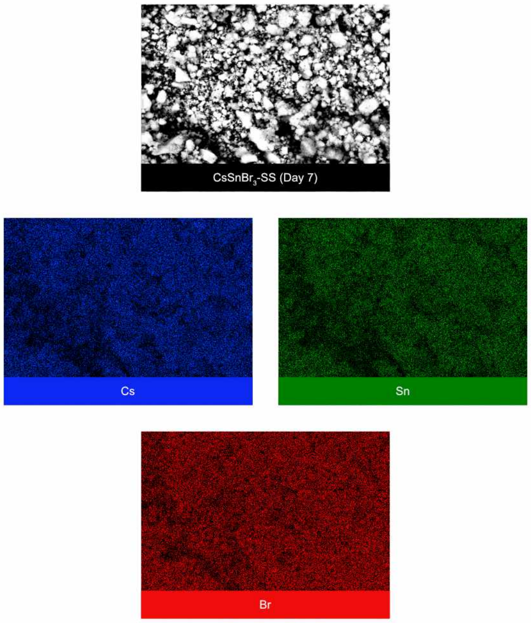

Figure S12. Elemental mapping of $CsSnBr_3$-SS after exposure to 65% relative humidity (Day 7).

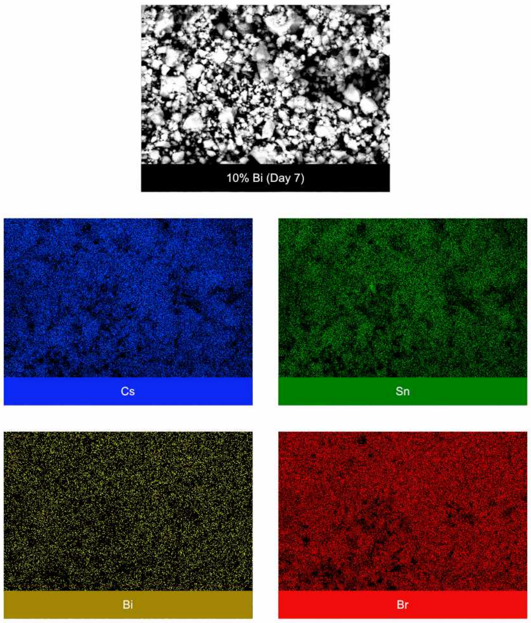

Figure S13. Elemental mapping of 10% Bi-substituted compound after exposure to 65% relative humidity (Day 7).
